# *Cj1199* Affect the Development of Erythromycin Resistance in *Campylobacter jejuni* through Regulation of Leucine Biosynthesis

**DOI:** 10.3389/fmicb.2017.00016

**Published:** 2017-01-17

**Authors:** Haihong Hao, Fei Li, Jing Han, Steven L. Foley, Menghong Dai, Xu Wang, Yulian Wang, Lingli Huang, Yawei Sun, Zhenli Liu, Zonghui Yuan

**Affiliations:** ^1^National Reference Laboratory of Veterinary Drug Residues and MOA Key Laboratory for Detection of Veterinary Drug Residues, Huazhong Agricultural UniversityWuhan, China; ^2^MOA Laboratory for Risk Assessment of Quality and Safety of Livestock and Poultry Products, Huazhong Agricultural UniversityWuhan, China; ^3^Hubei Collaborative Innovation Center for Animal Nutrition and Feed Safety, Huazhong Agricultural UniversityWuhan, China; ^4^Division of Microbiology, National Center for Toxicological Research, U.S. Food and Drug Administration, JeffersonAR, USA

**Keywords:** *Cj1199*, *C. jejuni*, biological function, leucine biosynthesis, erythromycin resistance

## Abstract

The aim of this study was to reveal the biological function of *Cj1199* which was overexpressed in the laboratory induced erythromycin resistant strains. The *Cj1199* deletion mutant (ΦCj1199) was constructed via insertional inactivation from its parent strain *Campylobacter jejuni* NCTC11168. The ΦCj1199 and NCTC11168 were then subjected to microarray and real-time PCR to find gene pathway of *Cj1199*. The antimicrobial susceptibility, antimicrobial resistance development, growth characteristics and leucine metabolism were examined to confirm the biological function of *Cj1199*. Our result showed that a total of 20 genes were down-regulated in ΦCj1199. These genes were mainly involved in leucine biosynthesis, amino acid transport and periplasmic/membrane structure. Compared to NCTC11168, ΦCj1199 was difficult to acquire higher-level erythromycin resistance during the *in vitro* step-wise selection. The competition growth and leucine-dependent growth assays demonstrated that ΦCj1199 imposed a growth disadvantage under pressure of erythromycin and in the leucine-free medium. In conclusion, *Cj1199* gene may directly regulate the leucine biosynthesis and transport and indirectly affect the development of erythromycin resistance in *C. jejuni*.

## Introduction

*Campylobacter jejuni* (*C. jejuni*), is an important foodborne pathogen whose infection often results in acute, self-limiting enteritis, but can lead to more serious complications such as Guillain-Barre Syndrome (Butzler, 2004; Hughes and Cornblath, 2005). Macrolides (mainly erythromycin), which can bind to the 50S subunit of bacterial ribosome and interfere with protein synthesis by inhibiting the elongation of peptide chains (Weisblum, 1995), are the valuable antimicrobials of choice to treat severe *Campylobacter* infections (Gibreel et al., 2005; EFSA, 2009). Point mutations in domain V of 23S rRNA (Taylor et al., 1997), in the ribosome protein L4/L22 and overexpression of eﬄux pumps are known as the major causes of resistance in *Campylobacter* (Lin et al., 2002; Gibreel et al., 2005). Despite the improved understanding of its general physiology and biochemistry, the molecular mechanisms involved in *Campylobacter* fitness and resistance development are largely unknown.

New approaches, like microarray and RNA-seq, have been used to determine the transcriptome changes associated with some gene mutations and the adaptive mechanisms of *C. jejuni* to erythromycin treatment (Chaudhuri et al., 2011; Xia et al., 2013). Our previous study, using a microarray analysis to find the transcriptional change involved in the development of macrolide resistance in *Campylobacter*, showed that a large number of genes were responsible to the development of macrolide resistance in *Campylobacter*, ranging from genes involved in energy metabolism to macromolecular metabolism to cell processes (Hao et al., 2013). In these differentially expressed genes, a gene of unknown function, *Cj1199*, was overexpressed in the erythromycin resistant *C. jejuni* mutants selected from their parent strains NCTC11168 and 81176. The expression level of *Cj1199* was dose dependent to the level of resistance in the series erythromycin resistant *C. jejuni* mutants selected from NCTC11168. Bioinformatic analyses suggested that *Cj1199* may encode putative iron/ascorbate-dependent oxidoreductase which may be involved in leucine biosynthesis and consequently affect the growth of the strains. Many previous studies revealed that leucine is a key amino acid of erythromycin resistant short peptides (E-Peptide) in *E. coli* (Dam et al., 1996; Tenson et al., 1997; Tripathi et al., 1998; Tenson and Mankin, 2001; Vimberg et al., 2004). Therefore, *Cj1199* may also be associated with E-Peptide mediated erythromycin resistance. The function of *Cj1199* on the development of macrolide resistance and fitness has yet to be determined. The objectives of this study were to decipher functionality and active network of this gene in *C. jejuni*.

## Materials and Methods

### Bacterial Strains and Growth Conditions

The *C. jejuni* NCTC11168 was kindly provided by Chinese Center for Disease Control. *C. jejuni* strains were routinely cultured on Mueller-Hinton (MH) agar (Oxoid, Basingstoke, UK) or blood agar plates at 42°C under microaerophilic conditions. *E. coli* DH5α was grown aerobically in Luria-Bertani medium at 37°C. The Ham’s F-12 nutrient powder mixture (SH30010, Thermo Fisher, Waltham, MA, USA) and Ham’s F-12m nutrient powder mixture (RR13033.01, Thermo Fisher, Waltham, MA, USA) lacking leucine were used for leucine biosynthesis test.

### Construction of *Cj1199* Deletion Mutant

A 2.5-Kb fragment containing *Cj1199* and its flanking region was amplified with primers of cj1199F and cj1199R (**Table 1**) by PCR and cloned into pGEM-T easy vector (Promega, Madison, WI, USA) to yield pL1199. A chloramphenicol resistance cassette (Cm^r^ marker) was amplified from pUOA18 using primers of ecatF and ecatR. Primers rF and rR were used to inversely amplify pL1199. The PCR product of the inverse amplification of pL1199 was digested with *Sma*I and *BamH*I and ligated with the Cm^r^ marker to yield plasmid pL1199T. The plasmid pL1199T was transformed into *E. coli* DH5α and then introduced to *C. jejuni* NCTC11168 by natural transformation (Jeon et al., 2010). The *Cj1199* deletion mutant (ΦCj1199) was selected on MH agar containing chloromycetin (20 μg/ml) and confirmed by PCR and DNA sequencing.

**Table 1:** Primers used for construction of Cj1199 mutant and for qRT-PCR

Primer name	Primer sequence	Product size (bp)
**Primers used for construction and confirmation of Cj1199 mutant**		
Cj1199F	TAGCGTATTTGATTTGCGTTTT	2536
Cj1199R	TATTTGCACCTATTCTTGGGTAA	
rF	ACCCCGGGAAATGGCTTAGTATACCCCCACT SmaI site	5140
rR	ACGGATCCGCTAGTATAACCTCTAAATTGAGG BamHI site	
ecatF	ACGGATCCAAAGAGTGACCGCCGAGA BamHI	810
ecatR	ACCCCGGGCAGTGCGACAAACTGGGA SmaI site	
16SF	CTGGCTCAGAGTGAACGC	533
16SR	CCCTTTACGCCCAGTGATT	
hipOF	AGAAGCCATCATCGCACCT	148
hipOR	TGCTGAAGAGGGTTTGGGT	
catF	AAAGAGTGACCGCCGAGATA	0/798^a^
catR	CAGTGCGACAAACTGGGATT	
Cj1199F	TGAACTTACCTATACTTGATT	954/1353^b^
Cj1199R	AAATACTTGCCACATCAG	
**Primers used for real-time qRT-PCR**		
cj0560F	GCATTTAGGTTTAGGCAAGAAG	159
cj0560R	GTCATCGTTTTAATCTCATCGCT	
cj0920F	CTATTGGTGTATTGGGTGTTGG	184
cj0920R	GCTTCAAACTGACCTCTTGGC	
cj1241F	AGTTACTGGAGTGCCGCTTG	147
cj1241R	AGATTGGTTGGGCTAGGATGT	
cj1388F	CAACTTCCTATCAACCCTGCTTC	221
cj1388R	GCACTTCTTGCTGGATAAGGAG	
cj1646F	CAGGCTGCTTACCAACTCG	275
cj1646R	GCATCGCTAATGGCAACG	
cj1717cF	GCACAGCAAAAGGCACAGG	232
cj1717cR	TCATCACTTCTTAAACTCTTCCAAT	
q16SF	GCTCGTGTCGTGAGATGTTG	199
q16SR	GCGGTATTGCGTCTCATTGTAT	

*^a^There should be no amplification product for NCTC11168, while 798 bp product for ΦCj1199; ^b^Product length of NCTC11168 should be 954 bp, product length of ΦCj1199 should be 1353 bp.*

### RNA Extraction

The cells of ΦCj1199 or *C. jejuni* NCTC11168 were grown in MH agar plate for 24 h at 42°C under microaerophilic conditions. For RNA extraction, RNAprotect Bacteria Reagent (Qiagen, Valencia, CA, USA) was added to the cultures immediately after the incubation to stabilize mRNA. The total RNA from each sample was extracted using the RNeasy Protect Mini Kit (Qiagen, Valencia, CA, USA) following the manufacture’s protocol. RNA samples were extracted from four independent experiments.

### DNA Microarray and Data Analysis

The differential gene expression between ΦCj1199 and *C. jejuni* NCTC11168 was identified using DNA microarray which was supplied by CapitalBio Corporation (Santa Clara, CA, USA). Briefly, iScript cDNA synthesis kit (BioRad, Hercules, CA, USA) was used for synthesis of cDNA from an RNA template via reverse transcription. The cDNA was then labeled with Cy5 or Cy3 dye. The labeled cDNA probes were co-hybridized onto one microarray slide (Roche NimbleGen 4 × 72K, Indianapolis, IN, USA). Hybridized slides were scanned using NimbleGen MS200, and the fluorescence intensities were collected with NimbleGen Scan Software. The linear normalization method was used for data analysis based on expression of the housekeeping genes. Normalized data was log transformed and loaded into MANOVA under R environment. Microarray spots with false discovery rate (FDR)-corrected *q*-values < 0.01 and fold change ≥2 in the *T*-test were regarded as differentially expressed genes. The differentially expressed genes were classified based on the genomic annotation in NCBI and then subjected to KEGG database for pathway analysis.

### Quantitative Reverse Transcriptase PCR (qRT-PCR)

The surplus cDNA of ΦCj1199 and *C. jejuni* NCTC11168 was subjected to real-time quantitative reverse transcriptase PCR (qRT-PCR) analysis to confirm the expression of some respective genes identified by DNA microarray. The primers (**Table 1**) of respective genes were designed using Primer 5 software. The qRT-PCR was performed using SYBR Green Ex Taq^TM^ kit (Takara, Madison, WI, USA) in IQ5 Multicolor Real-time PCR Detection System (Bio-Rad) following method described in previous study (Hao et al., 2010). The qRT-PCR was initiated with a 30 s denaturation at 95°C, followed by 38 cycles of amplification with 5 s of denaturation at 95°C, 30 s of annealing according to the melting temperatures of amplifications. The melting curve was performed from 65 to 95°C (1 s hold per 0.2°C increase) to check the specificity of the amplified product.

### Determination of Antimicrobial Resistance (AMR)

Minimum inhibitory concentrations (MICs) of azithromycin (AZM), erythromycin (ERY), tylosin (TYL), ciprofloxacin (CIP), nalidixic acid (NAL), olaquindox (OLA), florfenicol (FFC), doxycycline (DOX), gentamicin (GEN), and levofloxacin (LEV) against ΦCj1199 and *C. jejuni* NCTC11168 were determined with broth microdilution method following the guidelines of the Clinical and Laboratory Standards Institute (CLSI, 2006). The concentrations of the antimicrobial agents tested ranged from 0.01 to 16 μg/mL. The microdilution plates (96 well plates) were incubated at 42°C for 24 h and the MIC was determined as the lowest concentration where visible bacterial growth was inhibited at the end of the incubation period. *C. jejuni* ATCC 33560 was used as a quality control strain.

For the minimal bactericidal concentration (MBC) of drugs against *C. jejuni* strains, 100 μL from each well was successively diluted 1:10 in MH broth. Then 10 μL was spread on MH agar plates and incubated at 42°C for 24 h for colony forming unit (CFU) counting. The MBC was defined as the lowest drug concentration that resulted in a 99.9% reduction in the bacterial density. The final result was expressed as mean of five independent experiments.

The mutant prevention concentration (MPC) of drugs was determined by agar method (Blondeau et al., 2001). The inoculum of *C. jejuni* was concentrated to 10^10^ CFU/mL. Bacterial suspensions were inoculated on the agar plates containing serial dilutions of drugs and cultured for 96 h. The MPC was the lowest drug concentration on agar plates without bacterial growth under micro-aerobic conditions.

### Erythromycin Tolerance Tested by Flow Cytometry (FCM)

The concentration of bacteria was adjusted to a 0.5 McFarland density. Serial twofold dilutions of erythromycin were prepared with MH broth. The final concentrations of erythromycin ranged from 0.0625 to 8 μg/mL. A 100 μL aliquot of bacteria was added to 900 μL MH broth containing different concentrations of erythromycin and incubated at 42°C in a micro-aerobic condition for 3 h. Each dilution was then centrifuged and the supernatant was discarded. The pellet was washed twice with sterile PBS and propidium iodide (PI), a membrane-impermeable DNA-intercalating dye, was used to stain the erythromycin-treated bacterial cells. One milliliter of buffer and 5 μL of PI were added to each sample, and then incubated for 30 min at 4°C. FCM analysis was conducted on CyAn ADP^TM^ FC500 flow cytometer with Summit^TM^ software (Beckman Coulter, Miami, FL, USA). The intensity of fluorescence of 10,000 cells labeled with PI was analyzed to obtain the mean channel fluorescence (MCF).

### *In vitro* Selection of Erythromycin Resistance

ΦCj1199 and NCTC11168 were subjected to step-wise selection in MH agar plate containing erythromycin. In the first step of selection, cells were plated on MH agar containing 0.25 μg/mL erythromycin. After 3–5 days of incubation under microaerophilic condition at 42°C, colonies were selected and repeatedly incubated with same concentration of erythromycin up to five times. The mutants obtained from first-step selection were then subjected to next step selection by exposing the cells to a twofold increased concentration of erythromycin. The procedure of selection was repeated up to 10 times to obtain highly erythromycin-resistant mutants. All *in vitro*-selected mutants were subjected to MIC test using the broth microdilution method as described above.

### Single Growth Curve and Pair-Wise Competition Experiments

Growth curves of ΦCj1199 and *C. jejuni* NCTC11168 were measured individually and repeated three times. Briefly, a fresh *C. jejuni* culture was diluted 100-fold into 10 mL MH broth and incubated for up to 36 h. Viable *C. jejuni* counts were determined at 4, 12, 24, and 36 h.

Pair-wise competition experiments were used to estimate the growth rate of ΦCj1199 compared with *C. jejuni* NCTC11168 in the erythromycin containing (1/2 MIC) or drug-free MH broth. Briefly, the same densities of the parent and mutant cultures were prepared before experiments. Equal volumes (0.1 mL) of adjusted cultures were mixed into 10 mL of fresh medium with or without erythromycin. After 24 h incubation, 0.1 mL bacterial culture was then transferred to another 10 mL fresh medium. The passage was repeated up to 10 times and the initial and final concentrations of parent and mutant were determined by standard plate counting on MH plate with (for the mutant population) or without 10 μg/mL chloromycetin (for the total population), respectively. The assays were repeated three times with three technical replicates.

### Leucine-Dependent Growth Test

Cultures of ΦCj1199 and *C. jejuni* NCTC11168 were adjusted to the same densities at the beginning of the experiments. Equal volumes (0.1 ml) of adjusted cultures were added into 10 mL Ham’s F-12 and F-12m individually. After 48 h incubation under microaerophilic conditions at 42°C, 0.1 mL of bacterial culture was then transferred to another 10 mL of fresh medium. The passage was repeated up to five times. Viable *C. jejuni* counts were determined by dilution and spatula method on MH plates (ICC, 1992). The experiment was performed in triplicate.

## Results

### Differentially Expressed Genes in ΦCj1199

The 20 genes down-regulated in ΦCj1199 are shown in **Table 2**. Five genes (*leuA, leuB, leuC, leuD*, and *trpE*) were involved in amino acid biosynthesis. The *leuABCD* genes participated in leucine biosynthesis pathway. Five genes (*Cj0919c, Cj0920c, peb1A, pebC*, and *ceuC*) encoded transporters or binding proteins. The permease protein (Cj0919c-Cj0920c), substrate-binding protein (Peb1A), and ATP-binding protein (PebC) worked cooperatively in an ABC-type amino acid transport system. The *ceuC* encoded enterochelin uptake permease which was also transporters or binding protein. The Cj0246c was involved in signal transduction. Six genes (*Cj0560, Cj1356c, Cj0423, Cj0424, Cj0425*, and *Cj0200c*) encoded putative integral membrane or periplasmic proteins. Another three (*Cj1025c, Cj1722c*, and *Cj1388*) were hypothetical genes.

**Table 2:** Differentially expressed genes in the mutant of ΦCj1199 comparing to its parent strain NCTC 11168.

Function class	Gene name	Gene function	Fold change
Signal tranduction	*Cj0246c*	putative MCP-domain signal transduction protein	0.3624
Transporters/binding proteins	*Cj0919c*	putative ABC-type amino-acid transporter permease	0.4997
	*Cj0922c/pebC*	amino-acid ABC transporter ATP-binding protein	0.461
	*Cj0921c/peb1A*	bifunctional adhesin/ABC transporter aspartate/glutamate-binding protein	0.4597
	*Cj0920c*	putative ABC-type amino-acid transporter permease	0.3920
	*Cj1353/ceuC*	enterochelin uptake permease	0.4557
	*Cj1241*	putative MFS transport protein/Arabinose eﬄux permease	2.3237
Amino acid biosynthesis	*Cj1716c/leuD*	isopropylmalate isomerase small subunin	0.3996
	*Cj1717c/leuC*	isopropylmalate isomerase large subunin	0.378
	*Cj1718c/leuB*	3-isopropylmalate dehydrogenase	0.348
	*Cj1719c/leuA*	2-isopropylmalate synthase	0.3167
	*Cj0345/trpE*	putative anthranilate synthase componentI	0.4076
Cell envelop	*Cj0560*	putative MATE family transport protein	0.3564
	*Cj1356c*	putative integral membrane protein	0.4983
	*Cj0423*	putative integral membrane protein	0.4534
	*Cj0425*	putative periplasmic protein	0.4918
	*Cj0424*	putative acidic periplasmic protein	0.4483
	*Cj0200c*	putative periplasmic protein	0.4689
Hypothetical proteins and unknown function proteins	*Cj1025c*	hypothetical protein	0.4974
	*Cj1722c*	hypothetical protein	0.4727
	*Cj1388*	putative endoribonuclease L-PSP	0.3849

**FIGURE 1:**
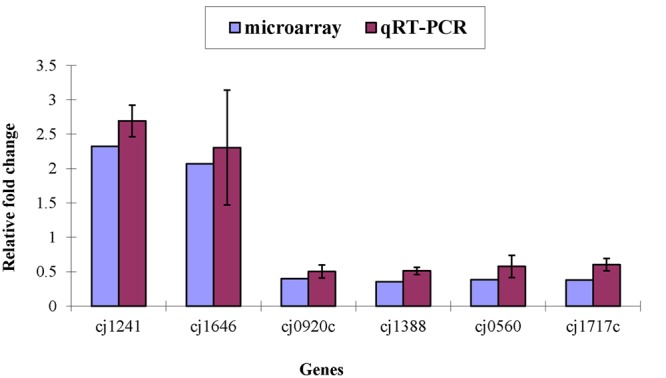
**Differential expression of target genes in ΦCj1199 and NCTC11168 determined by real-time qRT-PCR and microarray.** The genes were randomly selected. RNA used for real-time qRT-PCR and microarray were prepared in the same time.

Only *Cj1241* gene encoding a putative major facilitator superfamily (MFS) transport protein was up-regulated in ΦCj1199. Using qRT-PCR to verify the microarray results, *Cj1241* gene was confirmed to be up-regulated, while other four respective genes (*Cj0920c, Cj1388, Cj0560*, and *Cj1717c*) were confirmed to be down-regulated (**Figure 1**).

### Antimicrobial Susceptibility of ΦCj1199

The antimicrobial susceptibility of the parent and mutant strains are presented in **Table 3**. There was no significant difference between the MIC of 11 antimicrobials in the ΦCj1199 and its parent strain NCTC11168. Both of the strains exhibited very low MIC (MIC ≤ 0.5 μg/mL) to the 14, 15, and 16-membered ring macrolides (erythromycin, azithromycin, and tylosin). The MBC and MPC of five antimicrobials in ΦCj1199 were similar (erythromycin, azithromycin, tylosin, olaquindox, and cydox) with that in NCTC11168 (**Table 3**).

**Table 3:** Minimum inhibitory concentration (MIC)/minimal bactericidal concentration (MBC)/mutant prevention concentration (MPC) of wild-type and mutant strains to different types of drugs.

Drug	Strains					
	Mutant			NCTC 11168		
	MIC (μg/mL)	MBC (μg/mL)	MPC (μg/mL)	MIC (μg/mL)	MBC (μg/mL)	MPC (μg/mL)
ERY	0.125	0.25	0.25	0.125	0.125	0.25
TYL	0.5	1	1	0.5	1	1
AZI	0.015	/	0.031	0.015	/	0.031
OLA	0.25	0.5	0.5	0.25	0.5	0.5
CYA	0.25	0.5	0.5	0.25	0.5	0.5
FFC	0.5	/	/	0.5	/	/
GEN	0.125	/	/	0.125	/	/
NAL	8	/	/	8	/	/
CIP	0.062	/	/	0.062	/	/
LEV	0.062	/	/	0.062	/	/
DOX	0.125	/	/	0.125	/	/

*ERY, erythromycin; TYL, tylosin; AZM, azithromycin; FFC, florfenicol; GEN, gentamicin; NAL, nalidixic acid; CIP, ciprofloxacin; LEV, levofloxacin; DOX, doxycycline; OLA, olaquindox; CYA, cydox. The “/” means not detected.*

### Erythromycin Tolerance of ΦCj1199

**FIGURE 2:**
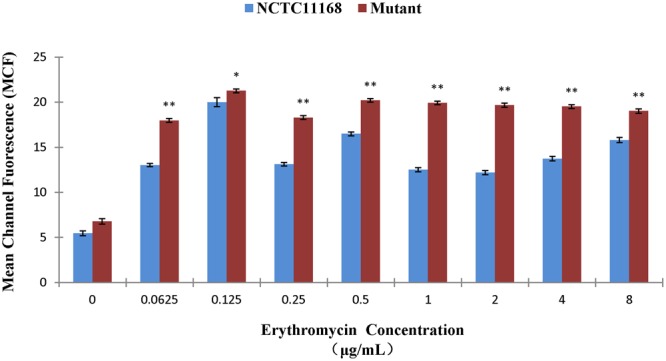
**Mean values of PI stain of ΦCj1199 and NCTC11168 cultured with different concentrations of erythromycin.** The asterisk (^∗^) and (^∗∗^) represent statistical significant difference with *P* ≤ 0.05 and *P* ≤ 0.01 comparing with *C. jejuni* NCTC11168, respectively.

The erythromycin tolerance of ΦCj1199 to increased concentration of erythromycin was detected by flow cytometer analysis. As shown in **Figure 2**, the mean value of the fluorescence intensity was increased following the increase of erythromycin concentration. However, when the concentration of erythromycin reached to their MIC (0.125 μg/mL), the mean PI intensity of *C. jejuni* NCTC11168 was significantly decreased, while the mean PI intensity of ΦCj1199 kept at its highest level. Under the treatment of different concentrations of erythromycin, the mean PI intensity of ΦCj1199 was higher than that of parent strain, which indicated that ΦCj1199 had higher mortality and lower tolerance to erythromycin.

### Development of Erythromycin Resistance *In vitro*

There was no significant difference between ΦCj1199 and NCTC11168 in the first and second steps of selections. When transferred to third step selection by 1 μg/mL erythromycin, ΦCj1199 descendants exhibited less growth than NCTC11168 descendants. When transferred to fourth step of selection, ΦCj1199 descendants could not grow on MH agar containing 2 μg/mL erythromycin, while *C. jejuni* NCTC11168 descendants grew well and successfully promoted to fifth step of selection by 4 μg/mL erythromycin. The erythromycin MIC of mutants selected from NCTC11168 was equal to or higher than 128 μg/mL, which was significantly higher than erythromycin MIC (4 μg/mL) of mutants selected from ΦCj1199.

### Growth Disadvantage of ΦCj1199

**FIGURE 3:**
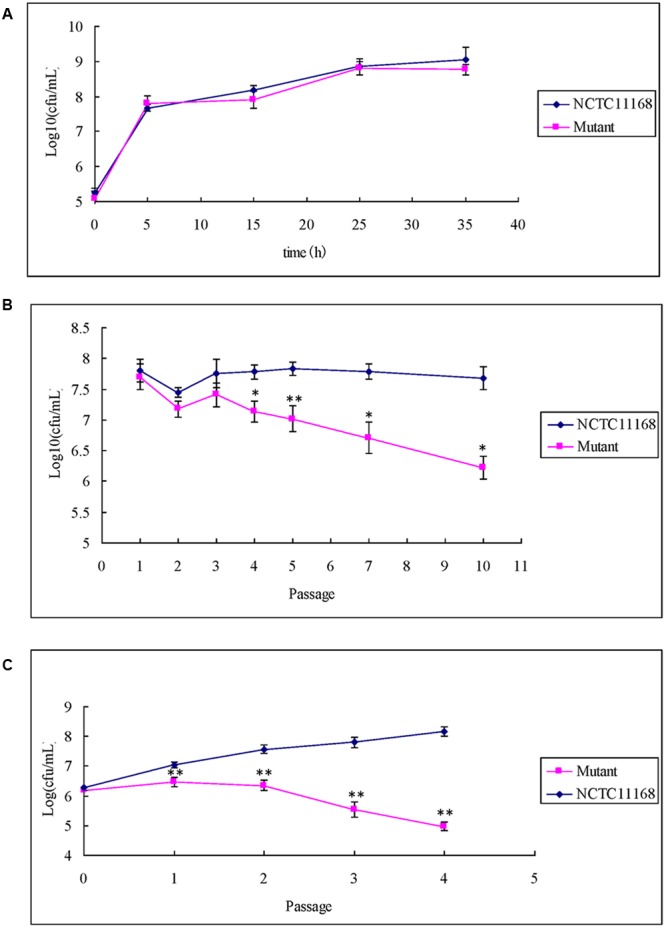
**Single growth curve and competitive growth of ΦCj1199 and NCTC11168 in the culture with or without erythromycin. (A)** Bacterial culture grown in MH for 36 h. Viable colonies were determined at 5, 15, 25, and 35 h. **(B,C)** Pair-wise competition of C. *jejuni* NCTC11168 and mutant was determined during 5–10 passages in the mixed culture. The Initial and final concentrations were determined by standard plate count on MH or MH with chloromycetin. The asterisk (^∗^) and (^∗∗^) represent statistical significant difference with *P* ≤ 0.05 and *P* ≤ 0.01 comparing with *C. jejuni* NCTC11168, respectively.

The ΦCj1199 and NCTC11168 were separately cultured in macrolide-free MH broth. No significant difference in growth rates was observed between the two strains in single cultures (**Figure 3A**).

When ΦCj1199 and NCTC11168 were mixed in pairs into drug-free MH broth, population of ΦCj1199 was decreased after the fifth passage and reduced by twofolds of magnitude after the 10th passage (**Figure 3B**).

In the mixture culture containing 1/2 MIC erythromycin, population of ΦCj1199 was reduced during passages. The ΦCj1199 was outcompeted by NCTC11168 in the drug-containing culture after the fourth passage (**Figure 3C**).

### Leucine-Dependent Growth

**FIGURE 4:**
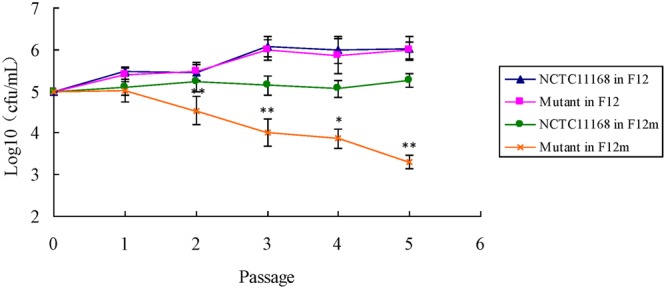
**The leucine-dependent growth test.** The growth curve of NCTC11168 and ΦCj1199 in Ham’s F12 and Ham’s F12m were determined. The asterisk (^∗^) and (^∗∗^) represent statistically significant difference with *P* ≤ 0.05 and *P* ≤ 0.01 comparing with *C. jejuni* NCTC11168, respectively.

There is no significant difference of the growth between ΦCj1199 and NCTC11168 in the medium F-12. Both of them grew well after five passages in the medium F-12 (**Figure 4**). However, the number of ΦCj1199 was remarkably decreased during the five times of passage in the medium F-12m. After the fifth passages in the medium F-12m, the number of ΦCj1199 mutant had been reduced by two orders of magnitude comparing with its parent strain NCTC11168 which kept at the level of 10^5^ CFU/mL. Conclusively, in absence of leucine, the growth of ΦCj1199 was much slower than NCTC11168.

## Discussion

To our knowledge, this is the first study of the function of *Cj1199* on the development of macrolide resistance and biological characteristics of *C. jejuni*. Previously, *Cj1199*, a gene of unknown function, was up-regulated in the macrolide resistant *C. jejuni* mutants selected by step-wise increased erythromycin *in vitro* from their parent strains NCTC11168 and 81176 (Hao et al., 2013). Bioinformatic analyses suggested that *Cj1199* may encode putative iron/ascorbate-dependent oxidoreductase. This gene was located in the downstream of *luxS* and over-expressed in the *luxS* null mutation (He et al., 2008). Therefore, the function of *Cj1199* attracted our attention. The gene knockout method, microarray assay, antimicrobial susceptibility test, FCM, and growth determination were used to find out the function and molecular mechanism of *Cj1199.*

From the microarray data, four genes (*Cj0921c*-*Cj0919c*-*Cj0920c*-*Cj0922c*) involved in amino acid transporters were obviously down-regulated in ΦCj1199. The *Cj0921*c gene was known to encode a bifunctional adhesin/ABC transporter aspartate/glutamate-binding protein (Peb1A; Pei and Blaser, 1993; Leon-Kempis Mdel et al., 2006). The Peb1A had higher affinity to both aspartate and glutamate, thus it could bind aspartate and glutamate as specific substrates to initiate the nutrient transport systems in gram-negative species (Pei and Blaser, 1993; Leon-Kempis Mdel et al., 2006). The glutamine could modulate bacterial metabolism of the arginine family as well as serine and aspartate families of amino acid and could reduce catabolism of most nutritionally essential amino acids including lysine, leucine, valine and serine, thus it may initiate signaling pathways related to amino acid metabolism in the intestinal bacteria (Dai et al., 2012, 2013). As shown in previous studies, *peb1A* was essential for the growth, invasion and intestinal colonization of *Campylobacter* species (Kervella et al., 1993; Pei et al., 1998; Leon-Kempis Mdel et al., 2006; Muller et al., 2007). Under a microaerobic environment, *peb1A* deficient mutants cannot grow on dicarboxylic amino acids which were the major nitrogen source (Leon-Kempis Mdel et al., 2006). In *C. jejuni*, the mutation in *peb1A* locus could reduce the interactions with epithelial cells and intestinal colonization of mice (Pei et al., 1998). There was no report about the characteristic of the other three genes (*Cj0919c*-*Cj0920c*-*Cj0922c*). The string 9.0 and KEGG analysis revealed that these four genes have close interaction with each other and they may work synergistically in the amino acid transport system. The gene cluster (*Cj0921c*-*Cj0919c*-*Cj0920c*-*Cj0922c*) may be essential for the uptake of nitrogen source which was indispensable for the growth and fitness of *Campylobacter* species. However, it is still unknown that how *Cj1199* effects the expression of *Cj0921c*-*Cj0919c*-*Cj0920c*-*Cj0922c* gene cluster.

The *leuABCD* gene cluster was involved in leucine biosynthesis (Labigne et al., 1992). The *leuA* encoded alpha-isopropylmalate synthase (alpha-IPMS) which was key enzyme to catalyze the first committed step in the leucine biosynthetic pathway (Yindeeyoungyeon et al., 2009). The *leuB* gene encoded 3-isopropylmalate dehydrogenase, while *leuC* and *leuD* genes encoded the large and small subunits of isopropylmalate isomerase, respectively (Tamakoshi et al., 1998). The *leuB* gene was reported to be essential for the survival in starvation, colony formation and growth in the Enterobacteriaceae (Matsumoto et al., 2011). In the sequences of *leuC* and *leuD*, cysteine residues for iron-sulfur binding and other amino acid residues involved in isomerase activity were highly conserved (Tamakoshi et al., 1998). The transcriptional derepresssion of a *leuC*-defective allele leaded to accumulation of Leu (+) mutations (Martin et al., 2011). Recent studies revealed that the *leuABCD* gene cluster also participated in the formation of norvaline and norleucine from 2-ketobutyrate and potentially contributed to non-polymeric carbon-chain elongation, which was essential in the synthesis of non-native metabolites in microorganisms (Sycheva et al., 2007; Shen and Liao, 2011). The coordinated expression of the functionally related *leuABCD* gene clusters may be mediated by LeuO and governed by transcription-driven DNA supercoiling (Chen et al., 2003; Wu and Fang, 2003). Our study indicated that the inactivation of *Cj1199* impacted the coordinated expression of *leuABCD* genes. However, the molecular mechanism involved in the regulation is still unknown. The leucine biosynthesis was versatile for many biogenic processes in prokaryotic and eukaryotic organisms. In the yeast and human cells, leucine biosynthesis regulated cytoplasmic iron-sulfur enzyme biogenesis in an atm1p (ABC transporter)-independent manner (Bedekovics et al., 2011). In many enteric bacteria and fungi, leucine, or a precursor of leucine can be used to regulate peripheral nitrogen metabolism with other cellular processes and can be selected directly or indirectly as a signal molecule in a wider metabolic context by very different mechanisms (Kohlhaw, 2003). Thus, leucine biosynthesis acts as a back door for effecting metabolism in cells (Kohlhaw, 2003).

Little is known about the gene cluster encoding putative membrane proteins (Cj0423-Cj0424-Cj0425). However, the *Cj0425* gene was considered to be an important contributor to the oxygen requirement and tolerance of *C. jejuni* (Kaakoush et al., 2007). The function of the other three genes involved in cell envelop (*Cj0560, Cj1356c*, and *Cj0200c*) is still unknown, but they may affect the fitness of ΦCj1199.

The down-regulation of the genes involved in amino acid transport and biosynthesis, and the genes encoding cell envelop may result in the disadvantage of nutrient utilization and growth in ΦCj1199. This hypothesis was confirmed by a series of competitive growth tests in the present study. Although there was no significant change in the short-term and single growth tests, the remarkable disadvantage of ΦCj1199 was observed in the long-term and competitive growth tests. Therefore, the *Cj1199* gene was essential for the fitness (growth) of *C. jejuni* by controlling the expression of the several genes involved in amino acid transport and leucine biosynthesis pathway.

According to the previous literature, a mechanism of macrolide resistance based on the expression of a specific short peptide in the cell was discovered several years ago (Tenson and Mankin, 1995; Tenson et al., 1996). The Shine-Delgarno region of the mini-gene encoding the short peptide was located in the 23S rRNA secondary structure (Tenson and Mankin, 2001). The peptide rendering *C. jejuni* resistance to erythromycin was one of the C-peptides. It bound erythromycin and removed the drug from its binding site of the ribosome and then sequestered it in an inactive complex (Tripathi et al., 1998). C-peptides showed a very strong bias in amino acid composition with a prevalence of the hydrophobic amino acid residues Leu, Ile, Val, Ala, Phe, and Trp. The most conserved position was the third, which was most commonly occupied by leucine. Therefore, it was presumable that the obstruction of leucine biosynthesis may lead to the failure of short peptide synthesis in the environment containing erythromycin. However, the role of *Cj1199* on the formation of C-peptides was not verified. No difference of the MIC between ΦCj1199 and NCTC11168 was observed in our study. Considering the limited sensitivity of MIC determination by broth microdilution method, the erythromycin susceptibility was detected by FCM, the result indicated that the tolerance of ΦCj1199 to erythromycin was decreased under the treatment by a series concentration of erythromycin. Additionally, ΦCj1199 showed remarkable disadvantage on the development of erythromycin resistance *in vitro* step-wise selection. Therefore, Cj1199 may not directly impact on the erythromycin resistance, but it can contribute to the initial survival rate of the bacteria during erythromycin treatment and may increase the chances of acquiring resistance.

## Conclusion

ΦCj1199 exhibited a weak competition of growth in a variety of competitive environments, especially in the medium with erythromycin. ΦCj1199 possessed lower risk for development of erythromycin resistance. Based on functions of differentially expressed genes, *Cj1199* has an impact on the biosynthesis of leucine and utilization of amino acid in gut, and thus may play an important role in the synthesis of short peptide and in the provision of energy and carbon. The genotype and phenotype change of ΦCj1199 indicates that *Cj1199* may contribute to the initial survival and colonization of the *C. jejuni* under the antibiotic treatment. *Cj1199* may impact the resistance acquirement or resistance development in a long term but not in a short term of growth. The role of *Cj1199* on the development of erythromycin resistance is indirectly dependent on the regulation of the expression of genes involved in amino acid biosynthesis and utilization. This study widened our understanding on the molecular mechanism of resistance and provides scientific reference for drug research and application.

## Ethical Statement

This article does not contain any studies with human participants or animals performed by any of the authors.

## Author Contributions

Conceived and designed the experiments: FL, HH, MD, XW, and ZY. Performed the experiments: FL, HH, YW, and YS. Analyzed the data: FL, HH, MD, JH, SF, LH, and ZY. Contributed reagents/materials/analysis tools: ZY, MD, HH, and ZL. Wrote the paper: HH, FL, JH, SF, and ZY.

## Conflict of Interest Statement

The authors declare that the research was conducted in the absence of any commercial or financial relationships that could be construed as a potential conflict of interest.
